# Trans-sexuality: Cultural issues involved in the management

**Published:** 2009

**Authors:** Devendra K. Gupta

**Affiliations:** Department of Paediatric Surgery, All India Institute of Medical Sciences, New Delhi-110 029, India

The term “trans-sexuality” needs to be differentiated from inter-sexuality. Intersexuality refers to a person with the ambiguity of genitalia, leading to undetermined sex. Trans-sexuality is a major problem that appears in childhood or in adult life with a permanent feeling of uneasiness and non-affiliation to the gender in which the person is born. A trans-sexual person has a strong feeling to look, behave, talk and experience the opposite sex, contrary to his own natural sex of assignment. The individual's brain refuses to accept his/her own identity. The incidence is one trans-sexual male in 30,000 adult men and one trans-sexual female in 100,000 adult women.[1] The socio-demographic profile is predominantly male (male/female sex ratio 3:2). Most cases, in the younger age group of less than 26 years, are referred for psychiatric help.[2]

Trans-sexuality has a variable geographical distribution. It was considered quite rare in developing countries about 15 years back. It is now showing a rising trend, though the incidence is still much less than seen in the West. In the paediatric age group, the problem of trans-sexuality is quite rare and often confused with intersex. Of the about 1200 intersex managed in the paediatric intersex clinic All India Institute of Medical Sciences, New Delhi, only a few cases of trans-sexuality were referred for advice.[3]

While some countries like the US and UK have separate laws providing identification to such individuals, in others, the individual being illiterate does not even understand his/her feelings and leads a forced life coping with familial pressures. In countries with strong cultural ties like those in India, the existence of trans-sexuality had not been recognized well due to cultural taboos and immense social pressure. Even if the victim had overcome the social pressures in search of an identity, the majority may land up being unisexual, physically heterosexual and mentally homosexual.

The aetiology is unknown. However, it may have resulted due to imbalance of hormonal activity during the prenatal and early perinatal phase of human development, affecting wrong sexual organization of the brain. A study has shown that though the regional grey matter variation in male to female trans-sexual is more similar to the pattern found in men than in women, they showed a significantly larger volume of regional grey matter in the right putamen compared to men, providing evidence that trans-sexuality is associated with distinct cerebral pattern, supporting the hypothesis that brain anatomy plays a role in gender identity.[4]

The management is an exceptionally difficult task which demands cooperation of a mental health professional (psychiatrist, psychologist), endocrinologist and a surgeon. “A trans-sexual, if not endured, has to be cured surgically and rehabilitated socially.” After having an appropriate approval from the competent authorities for the change of sex, the treatment includes sex-reassignment surgery, gender specific hormone therapy and social rehabilitation, keeping a watch for the known risks and treatment related complications. The results following male genitoplasty, including phalloplasty, still remain challenging. Most of the converted males remain inadequate despite insertion of penile prosthesis. However, it is the change in the mental status that they appreciated most.[5,6] Life becomes meaningful if they find like minded partners with the mutual understanding. On the contrary, it is much easier to achieve female genitoplasty and convert males to females. Various types of vaginoplasties can be performed (skin, ileum, and colon). However the long term postoperative results are better with the use of vascularised segment of the bowel (ileum or the sigmoid colon). Trans-sexual women (male to female) function much better physically, emotionally, psychologically and socially, while the male trans-sexual (female to male) has problems with partner relationship and sexuality.[7]

The quality of life is poor after sex re-assignment. It requires a lot of re-adjustments in the general life with physical and personal limitations.[8] Not only medical but also the social and the ethical consequences of the treatment need careful considerations before subjecting the individual to change of sex. The trans-sexual needs empathy and support. In developing countries, with strong social-cultural traditions, the problems related to the schooling, sex identity, inheritance, paternity and job opportunities are much more compounded in trans-sexual individuals and need to be addressed at the appropriate stage with government and non-governmental support. To circumvent child bearing issues and keep the mind of the trans-sexual gainfully occupied, child adoption is strongly recommended since it helps in coping with stress and allows life to proceed happily.
